# Glycemic markers and relation with arterial stiffness in Caucasian subjects of the MARK study

**DOI:** 10.1371/journal.pone.0175982

**Published:** 2017-04-17

**Authors:** Leticia Gomez-Sanchez, Luis Garcia-Ortiz, Maria C. Patino-Alonso, Jose I. Recio-Rodriguez, Natalia Feuerbach, Ruth Marti, Cristina Agudo-Conde, Emiliano Rodriguez-Sanchez, Jose A. Maderuelo-Fernandez, Rafel Ramos, Manuel A. Gomez-Marcos, for the MARK Group

**Affiliations:** 1 Primary Care Research Unit, the Alamedilla Health Center, Castilla and León, Health Service (SACyL) Salamanca, Spain; 2 Biomedical Research Institute of Salamanca (IBSAL), Salamanca, Spain; 3 Biomedical and Diagnostic Sciences Department, University of Salamanca, Salamanca, Spain; 4 Statistics Department, University of Salamanca, Salamanca, Spain; 5 Department of Nursing and Physiotherapy, University of Salamanca, Salamanca, Spain; 6 San Agustín Health Center, Illes Balears Health Service (IBSALUT), Palma of Mallorca, Spain; 7 Unitat of Suport the Recerca of Girona. Institut Universitari d’Investigacio in Atenció Primària Jordi Gol (IDIAP Jordi Gol), Girona, Spain; 8 Institut d’Investigació Biomèdica of Girona Dr. Josep Trueta (IDBGI). Girona, Spain; 9 Department of Medicine, University of Salamanca, Salamanca, Spain; 10 Departament of Ciències Mèdiques, Facultat of Medicina. Universitat of Girona, Girona, Spain; 11 MARK Group. redIAPP: Research Network in Preventive Activities and Health Promotion, Girona, Spain; Universita degli Studi Magna Graecia di Catanzaro Scuola di Medicina e Chirurgia, ITALY

## Abstract

**Background:**

Effect of prediabetes and normal glucose on arterial stiffness remains controversial. The primary aim of this study was to investigate the relationship of fasting plasma glucose (FPG), postprandial glucose (PG) and glycosylated haemoglobin (HbA1c) with brachial-ankle pulse wave velocity (baPWV) and cardio-ankle vascular index (CAVI) in Caucasian adults. The secondary aim was to analyse this relationship by glycaemic status.

**Methods:**

Cross-sectional study. Setting: Primary care. Participants: 2,233 subjects, 35–74 years. Measures: FPG (mg/dL) and HbA1c (%) of all subjects were measured using standard automated enzymatic methods. PG (mg/dL) was self-measured at home two hours after meals (breakfast, lunch and dinner) for one day using an Accu-chek ^®^ glucometer. CAVI was measured using a *VaSera VS-1500*^®^ device *(Fukuda Denshi*), and baPWV was calculated using a validated equation.

**Results:**

CAVI and baPWV values were significantly higher in subjects with diabetes mellitus than in glucose normal and prediabetes groups (p<0.001). FPG, PG and HbA1c were positively associated with CAVI and baPWV. The β regression coefficient for: HbA1c was 0.112 (CI 95% 0.068 to 0.155) with CAVI, 0.266 (CI 95% 0.172 to 0.359) with baPWV; for PG was 0.006 (CI 95% 0.004 to 0.009 and for FPG was 0.005 (CI 95% 0.002 to 0.008) with baPWV; and for PG was 0.002 (CI 95% 0.001 to 0.003) and 0.003 (CI 95% 0.002 to 0.004) with CAVI (p<0.01 in all cases). When analysing by hyperglycaemic status, FPG, PG and HbA1c were positively associated with CAVI and baPWV in subjects with type 2 diabetes mellitus.

**Conclusion:**

FPG, PG and HbA1c show a positive association with CAVI and baPWV, in Caucasian adults with intermediate cardiovascular risk factors. When analysing by hyperglycaemic status, the association is only maintained in subjects with type 2 diabetes mellitus.

**Trial registration:**

Clinical Trials.gov Identifier: NCT01428934. Registered 2 September 2011. Retrospectively registered. Last updated September 8, 2016.

## Introduction

A positive relationship between glycosylated haemoglobin (HbA1C), postprandial glucose (PG) and fasting plasma glucose (FPG) with cardiovascular morbidity in individuals with type 2 diabetes mellitus has been shown in several studies [1–3]. Arterial stiffness is an independent predictor of mortality in both the general population and in diabetics. [4].

In subjects with normal blood glucose metabolism, the association of FPG, PG and HbA1c with arterial stiffness is unclear. Studies have found an association between brachial-ankle pulse wave velocity (baPWV) and FPG [5], between cardio-ankle vascular index (CAVI) and PG [6] and between arterial stiffness and HbA1c [7–9]. However, other studies have not found an association between carotid-femoral pulse wave velocity (cfPWV) and FPG, PG [9] or HbA1c [10].

In subjects with prediabetes who were diagnosed based on their impaired fasting glucose (IFG), baPWV was associated with FPG and HbA1c [11–13]. However, the finding of increased arterial stiffness in prediabetes was not supported in all studies. Thus, in a study by Asklepios [14] involving 1,927 subjects, after controlling for age, gender and mean blood pressure, IFG was not found to be associated with arterial stiffness. Li et al. [15] showed that subjects with IFG and impaired glucose tolerance (IGT) exhibit higher baPWV. This was not seen in subjects who only presented with IFG. Consequently, the relationship between prediabetes and arterial stiffness requires further research.

Multiple studies have demonstrated that arterial stiffness is greater in subjects with diabetes than in people without diabetes [16, 17]. Likewise, better blood glucose control along with reduced blood pressure lessens or prevents the progression of aortic stiffness in patients with type 2 diabetes mellitus [18]. It is important to keep in mind that the independent association between arterial stiffness and type 2 diabetes mellitus has not been proven consistently across all studies. In a review conducted by Cecelja et al. [19], diabetes mellitus was independently associated with cfPWV in 52% of the reported studies.

Based on these data, we can conclude that there is not a clear association between dysglycaemia markers in subjects with normal glucose metabolism, prediabetes and type 2 diabetes mellitus and arterial stiffness. In subjects with intermediate cardiovascular risk, it is important to analyse new cardiovascular risk factors and the association between these factors for personalized risk stratification. In this group of patients, the association of glycaemic markers with arterial stiffness has not been studied; therefore, we designed our study with the following objectives. The primary aim of this study was to investigate the relationship of FPG, PG and HbA1c with CAVI and baPWV in Caucasian adults with intermediate cardiovascular risk. The secondary aim was to analyse this relationship by glycaemic status.

## Methods

This trial is a cross-sectional study of subjects recruited to the *improving interMediAte RisK management (MARK)* study (NCT01428934) [20], which is a longitudinal study designed to assess whether the ankle-brachial index, arterial stiffness (measured by CAVI and baPWV), postprandial glucose, glycosylated hemoglobin, self-measured blood pressure, and the presence of comorbidities are independently associated with the occurrence of vascular events. It also investigates whether the predictive capacity of current risk equations can be improved in the intermediate risk population. The current study focuses on the baseline visit. The second step will be a 5- and 10-year follow-up of the cohort to assess cardiovascular morbidity and mortality.

The study was approved by the Research Ethics Committees of the Primary Care Research Institute Jordi Gol, Health Care Area of Salamanca and Palma of Mallorca. All participants gave their written informed consent before data collection.

### Study population

**Fig 1:**
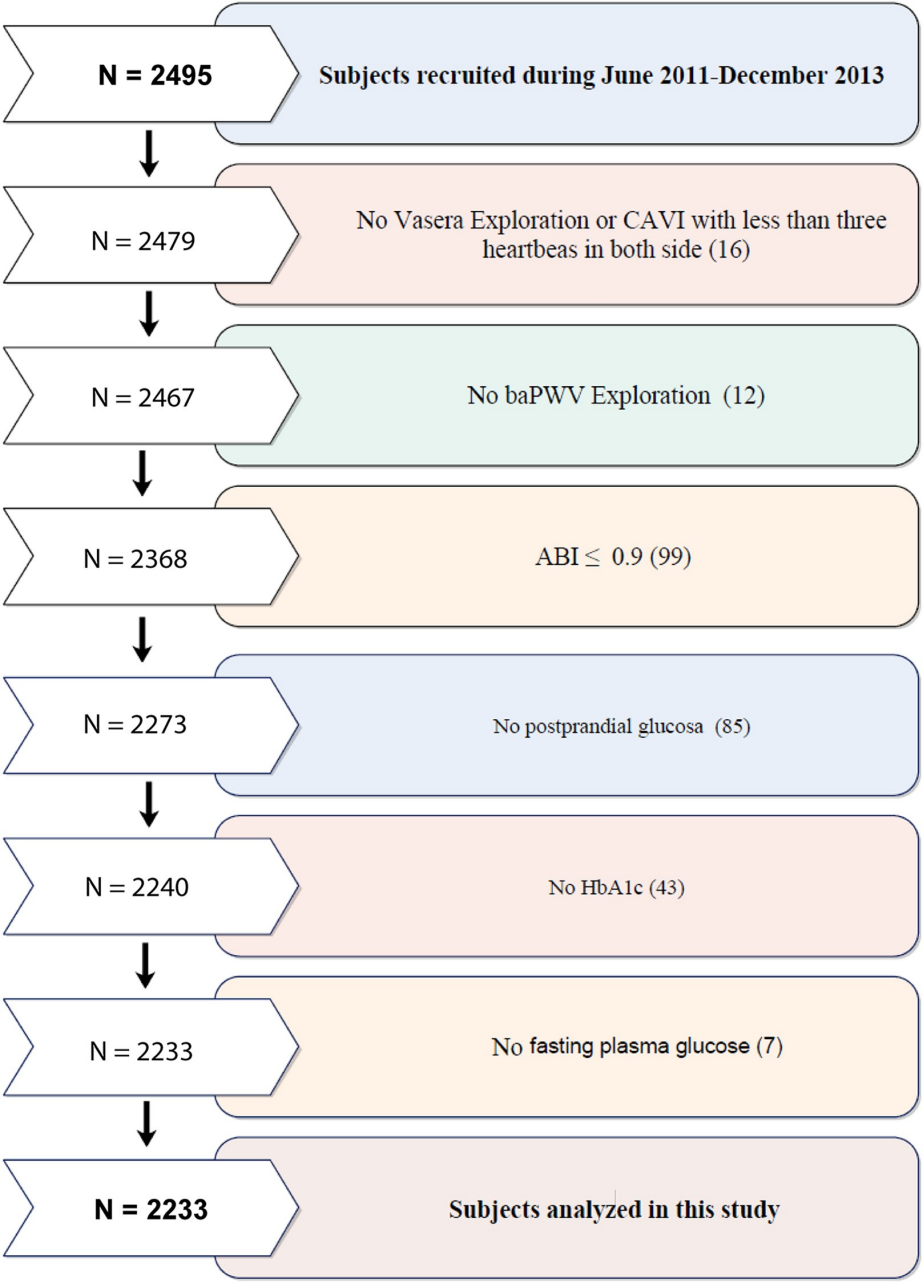
Flow chart of this MARK substudy. N, number; CAVI, cardio-ankle vascular index; baPWV, brachial-ankle pulse wave velocity; ABI, ankle-brachial index; HbA1c, glycosylated hemoglobin.

In this multicenter project, study population selection was performed by random sampling from individuals who met the inclusion criteria and were seeing general practitioners from July 2,011 to June 2,013 at 6 primary care centers from three Spanish Autonomous Communities. Subjects were recruited from those aged 35 to 74 years with intermediate cardiovascular risk defined as 10-year coronary risk ranging from 5%–15% according to the adapted Framingham risk equation [21]; 10-year vascular mortality risk ranging from 1%–5% according to the scoring risk in Europeans equation [22]; or moderate risk according to the European Society of Hypertension guidelines for the management of arterial hypertension [23]. Exclusion criteria included end-stage disease or institutionalization at the time of the visit or history of atherosclerotic disease. This study analysed 2,233 of the 2,495 recruited in MARK study, the causes of exclusion are shown in Fig 1. This study included 854 subjects defined as having normal glucose, 756 subjects classified as having prediabetes and 623 with type 2 diabetes mellitus. We have adapted cut-offs of HbA1c, mean 2-hour postprandial glucose and normal glucose in accordance with current American Diabetes Association criteria [24]. Normal glucose was defined as FPG < 100 mg/dL, mean 2-h PG < 140 mg/dL, HbA1c < 5.7% and not taking antidiabetic drugs. Prediabetes was defined as FPG values between 100 and 125 mg/dL or mean 2-h PG between 140 and 199 mg/dL or HbA1c between 5.7% and 6.4% and not taking antidiabetic drugs. Type 2 diabetes mellitus was defined as FPG ≥ 126 mg/dL or mean 2-h PG ≥ 200 mg/dL or HBa1c ≥ 6.5% or receiving treatment with antidiabetic drugs.

All participants were informed of the objectives and procedures of the study and signed informed consent form to participate. The study has been approved by the Clinical Research Ethics Committee of the Primary Care Research Institute Jordi Gol, the Health Care Area of Salamanca and Palma of Mallorca. The study was conducted following the recommendations of the Declaration of Helsinki [25]. The confidentiality of information provided by participants was ensured, complying with the rules established by Spanish Organic Law 15/1999, of 13 December on the Protection of Personal Data.

### Variables and measurement instruments

A detailed description of procedures for clinical data collection, anthropometric measurements, and laboratory tests has been published elsewhere [20].

### Laboratory data

Venous blood sampling was performed between 08:00 and 09:00 after the individuals had fasted and abstained from smoking and the consumption of alcohol and caffeinated beverages for the previous 12 hours. FPG (mg/dl) and HbA1c (%) were also be determined using standard enzymatic automated methods. PG (mg/dl) was self-measured by subjects at home 2 hours after meals (breakfast, lunch and dinner) for one day using an Accuchek ^®^ glucometer (Roche Diagnostics Corporation, Spain). PG was calculated as the average of the three measurements. Cholesterol and triglycerides concentration were determined by enzymatic methods and high density lipoprotein cholesterol after apo B containing lipoprotein precipitation. Low density lipoprotein cholesterol was determined by the Friedewald formula. Atherogenic index was determined by the formula (atherogenic index = total cholesterol/HDL cholesterol).

### Cardio-Ankle Vascular Index (CAVI) and brachial-ankle Pulse Wave Velocity (baPWV)

CAVI was measured using a *VaSera VS-1500*^®^ device *(Fukuda Denshi*) [26, 27]. CAVI values are calculated automatically by estimating the stiffness parameter β with the following equation: β = 2ρ x 1 / (Ps − Pd) x ln (Ps / Pd) x PWV^2^, where ρ is blood density, Ps and Pd are systolic blood pressure and diastolic blood pressure in mmHg, and PWV is measured between the aortic valve and the ankle [28]. The mean coefficient of variation of CAVI measurement is less than 5%, which is small enough to allow for clinical use of the index and confirms that CAVI has a favorable reproducibility [27]. Abnormal CAVI (≥ 9) represents subclinical atherosclerosis [28–32].

baPWV was estimated using the equation, baPWV = (0.5934 × height (cm) + 14.4724)/tba (tba is the time interval between the arm and ankle waves) [33]. Measurements were performed with the patient in supine position after resting for 10 minutes in a quiet room at a stable temperature. Subjects were instructed not to smoke or practice exercise in the hour prior to the test.

### Anthropometric measurements

Body weight was measured twice with a certified electronic scale (Seca 770, Medical scale and measurement systems, Birmingham, United Kingdom) after adequate calibration (precision ± 0.1 kg). Readings were rounded to 100 g. Height was measured with a stadiometer (Seca 222), and the average of two measurements was recorded. Body mass index was calculated as weight (kg) divided by height squared (m^2^). Waist circumference was measured according to the 2007 recommendations of the Spanish Society for the Study of Obesity [34]. All measurements were performed with the subjects standing, wearing no shoes, and in light clothing.

#### Office or clinical blood pressure

Office blood pressure measurement involved three measurements of systolic blood pressure and diastolic blood pressure with a validated OMRON model M10-IT sphygmomanometer (Omron Health Care, Kyoto, Japan). The measurements followed the recommendations of the European Society of Hypertension [35], and the averages of the last two measurements were used. PP was defined as SBP—DBP. Mean arterial pressure (MAP) was calculated as [(2 X DBP) + SBP]/3.

#### Tobacco

Smoking history was assessed by asking questions about the participant’s smoking status (smoker/non-smoker). We considered smokers to include those who currently smoke or who have stopped smoking within the past year.

#### Alcohol

consumption was assessed through a structured questionnaire and was expressed in grams per week.

The researchers who performed the different tests were blinded to the clinical data of the subjects. All assessments were made within a period of 10 days.

### Statistical analysis

Results are expressed as the mean ± standard deviation for quantitative variables or as the frequency distribution for qualitative variables. ANOVA with Bonferroni post hoc tests were used to identify significant differences in continuous variables among unadjusted group means. Additionally, χ^2^ tests were used to analyse differences in categorical variables among groups. Multiple linear regression models (ENTER method) were used to analyse the associations of HbA1c, PG and FPG, independent variables with CAVI and baPWV as the dependent variables. We used two models: Model 1 adjusted for age (years) and gender (0 = male and 1 = female), while Model 2 adjusted for age (years), gender (0 = male and 1 = female), smoking (0 = Not and 1 = Yes), alcohol consumption (gr/week), body mass index, mean arterial pressure, atherogenic index, antihypertensive drugs (0 = No and 1 = Yes), antidiabetic drugs (0 = No and 1 = Yes) and lipid lowering drugs (0 = No and 1 = Yes). The analysis was performed with the overall sample and in groups of subjects with normal glucose, prediabetes and diabetes mellitus. The marginal means in the three groups that were analysed for CAVI and for baPWV were estimated using ANCOVA, after adjusting for confounding variables used in model 2 of the multiple regression. Logistic regression analysis was performed for glycaemic status using baPWV (< 15 = 0 and ≥ 15 = 1) and CAVI (< 9 = 0 and ≥ 9 = 1) as dependent variables, HbA1c, PG and FPG as independent variables, and the variables used in model 2 as adjustment variables. Data were analysed using SPSS Statistics for Windows, Version 23.0 (IBM Corp, Armonk, NY). Values of p<0.05 were considered statistically significant.

## Results

The mean age of the 2,233 subjects enrolled in this study was 61.4 ± 7.6 years, and 1,385 subjects (62%) were males. Among all subjects, the mean FPG was 108 ± 35 mg/dL, the mean PG was 120 ± 38 mg/dL and the mean HbA1c was 6.1 ± 1.1. The mean CAVI was 8.8 ± 1.2, and the mean baPWV was 14.9 ± 2.5 m / sec (Table 1).

**Table 1:** Characteristics of study participants.

Variables	Participants n = 2233
	Mean±SD/n° (%)
Age (years)	61.4±7.6
Sex Males n (%)	1385 (62.0)
Smoking n (%)	1630 (73.0)
Alcohol consumption (gr/week)	73.0±118.2
BMI (kg/m^2^)	29.3±4.4
BMI ≥ 30 n (%)	819 (36.7)
Waist circumference (cm)	101.1±11.5
SBP (mmHg)	137.1±17.3
DBP (mmHg)	84.4±10.2
Pulse pressure (mmHg)	52.8±14.1
Mean arterial pressure (mmHg)	101.9 ± 11.2
Hypertension n (%)	1624 (72.7)
Antihypertensive drugs n (%)	1148 (51.4)
Total Cholesterol (mg/dl)	225.4±40.9
LDL Cholesterol (mg/dl)	140.1±34.9
HDL Cholesterol (mg/dl)	49.8±12.9
Triglycerides (mg/dl)	145.3±96.2
Atherogenic index	4.8±1.3
Dyslipidemia n (%)	1498 (67.1)
Lipid lowering drugs n (%)	639 (28.6)
FPG (mg/dl)	107.9±34.6
HbA1c	6.1±1.1
Postprandial glucose (mg/dl)	120.3±38.2
Antidiabetic drugs n (%)	461 (20.6)
CAVI	8.8±1.2
CAVI ≥ 9 n (%)	1013 (45.4)
baPWV (m/s)	14.9±2.5
baPWV ≥15 (m/s) n (%)	931 (41.7)

Values are means (standard deviations) for continuous data and number and proportions for categorical data.

SD Standard deviation. BMI body mass index. SBP Systolic blood pressure. DBP Diastolic blood pressure. LDL low density lipoprotein. HDL high density lipoprotein. FPG fasting plasma glucose. HbA1c glycosylated hemoglobin. CAVI cardio-ankle vascular index. baPWV brachial-ankle pulse wave velocity.

Table 2 shows a comparison of characteristics among subjects classified into three groups: normal glucose, prediabetes and type 2 diabetes mellitus. All the variables analysed except sex, diastolic blood pressure, mean arterial pressure and prevalence of dyslipidemia differed significantly among these groups. Body mass index, waist circumference, triglycerides, FPG, PG, HbA1c, baPWV and antihypertensive drugs increased with deterioration of glucose status. However, there are no differences in mean CAVI between subjects presenting normal metabolism and prediabetic individuals.

**Table 2:** Characteristics of study participants by diabetes status.

Variables	Normal glucose n = 854 (38.2)	Prediabetes n = 756 (33.9)	Type 2 DM n = 623 (27.9)	P value
Age (years) * ^¥^	60.5±8.0	62.1±62.1	62.0±7.4	<0.001
Sex males n (%)	573 (62.9)	484 (64.0)	364 (58.4)	0.108
Smoking n (%) ^¥^ ^#^	256 (30.0)	213 (28.2)	134 (21.5)	<0.001
Alcohol consumption (gr/week) ^¥^ ^#^	78.6±120.0	77.3±122.0	59.9±109.9	0.005
BMI (kg/m^2^) * ^¥^ ^#^	28.1±3.9	29.6±4.2	30.5±4.9	<0.001
BMI ≥ 30 n (%)*^¥^^#^	218 (25.5)	294 (38.9)	307 (49.3)	<0.001
Waist circumference (cm) * ^¥^ ^#^	97.8±10.7	102.2±11.2	104.1±12.1	<0.001
SBP (mmHg) ^¥^	135.6±17.6	137.2±17.4	139.1±16.6	0.001
DBP (mmHg)	84.5±10.5	84.4±10.2	84.1±9.8	0.635
Pulse pressure (mmHg) ^¥^ ^#^	51.1±13.5	52.7±14.4	55.1±14.1	<0.001
Mean arterial pressure (mmHg)	101.5 ± 11.7	102.0 ± 11.1	102.4 ± 10.6	0.363
Hypertension n (%)	593 (69.4)	549 (72.6)	482 (77.4)	0.001
Antihypertensive drugs n (%)* ^¥^ ^#^	362 (42.4)	392 (51.9)	394 (63.2)	<0.001
Total Cholesterol (mg/dl) ^¥^ ^#^	232.1±38.6	231.7±39.7	208.4±40.5	<0.001
LDL Cholesterol (mg/dl) ^¥^ ^#^	146.5±33.5	145.9±33.5	124.1±34.9	<0.001
HDL Cholesterol (mg/dl) * ^¥^	51.6±14.2	49.1±11.7	48.1±12.1	<0.001
Triglycerides (mg/dl) * ^¥^ ^#^	132.7±78.9	145.1±78.2	162.7±129.1	<0.001
Atherogenic index ^¥^ ^#^	4.8±1.2	4.9±1.3	4.5±1.3	<0.001
Dyslipidemia n (%)	585 (68.5)	503 (66.5)	410 (65.8)	0.263
Lipid lowering drugs n (%) ^¥^ ^#^	187 (21.9)	189 (25.0)	263 (42.2)	<0.001
FPG (mg/dl) * ^¥^ ^#^	87.8±7.6	100.3±11.3	144.7±45.9	<0.001
HbA1c * ^¥^ ^#^	5.3±0.3	5.8±0.3	7.5±1.4	<0.001
Postprandial glucose (mg/dl) * ^¥^ ^#^	102.1±13.6	111.2±20.4	156.3±51.1	<0.001
CAVI ^¥^ ^#^	8.7±1.1	8.8±1.1	9.1±1.2	<0.001
CAVI ≥ 9 n (%) ^¥^ ^#^	345 (40.4)	332 (43.9)	336 (53.9)	<0.001
baPWV (m/s) * ^¥^ ^#^	14.4±2.3	14.8±2.3	15.6±2.7	<0.001
baPWV ≥15 (m/s) n (%) ^¥^ ^#^	302 (35.4)	307 (40.6)	322 (51.7)	<0.001

Values are means (standard deviations) for continuous data and number and proportions for categorical data.

Differences among groups: continuous variables analysis of variance and post hoc using the Bonferroni tests. Categorical variables using the Chi-square test.

DM diabetes mellitus. BMI body mass index. SBP Systolic blood pressure. DBP Diastolic blood pressure. LDL low density lipoprotein. HDL high density lipoprotein. FPG fasting plasma glucose. HbA1c glycosylated hemoglobin. CAVI cardio-ankle vascular index. baPWV brachial-ankle pulse wave velocity.

*p value < 0.05 between normal and pre-diabetes.

^¥^p value < 0.05 between normal and diabetes.

^#^p value < 0.05 between pre-diabetes and diabetes.

**Fig 2:**
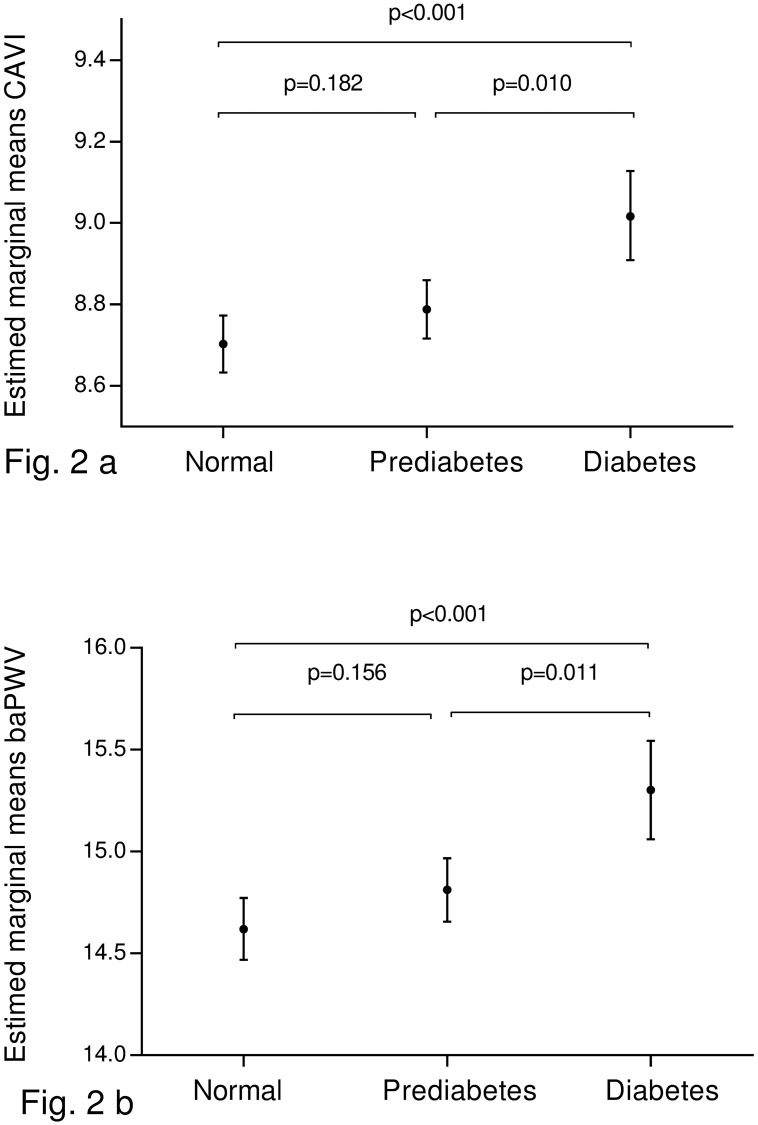
Estimated averages and standard deviations of CAVI (a) and baPWV (b) in diabetic subjects, prediabetic subjects and subjects with normal blood glucose. Adjusted for Age (years). Gender (0 = male and 1 = female). Smoking (0 = Not and 1 = Yes). Body mass index. Mean arterial pressure. Atherogenic index. Alcohol consumption. Antihypertensive drugs (0 = Not and 1 = Yes). Antidiabetic drugs and lipid lowering drugs (0 = Not and 1 = Yes). Differences among groups: analysis of variance and post hoc using the Bonferroni tests. CAVI cardio-ankle vascular index. baPWV brachial-ankle pulse wave velocity.

Fig 2 shows the estimated marginal means of CAVI and baPWV values among subjects with different glycaemic statuses. After adjusting for cardiovascular risk factors and concomitant medications, the mean values of CAVI and baPWV were significantly higher in subjects with diabetes than in those with normal glucose or prediabetes (p < 0.05). There were no differences in CAVI and baPWV between subjects with normal glucose and prediabetes.

Table 3 shows multiple linear regression analysis for the overall sample. FPG, PG and HbA1c were positively associated with CAVI and baPWV. In model 2, the β regression coefficient of HbA1c was 0.112 with CAVI and 0.266 with baPWV. The β coefficient values of PG was 0.006 and of FPG was 0.005 with baPWV and 0.002 and 0.003 with CAVI, respectively (p<0.01 in all cases).

**Table 3:** Multiple regression analysis of HbA1c, PG and FPG with CAVI and baPWV.

	Dependent variable					
	CAVI			baPWV		
Model 1	β	95% CI	p	β	95% CI	p
HbA1c	0.103	0.068 to 0.137	<0.001	0.388	0.309 to 0.466	<0.001
PG	0.002	0.001 to 0.003	<0.001	0.010	0.008 to 0.013	<0.001
FPG	0.003	0.002 to 0.004	<0.001	0.011	0.009 to 0.014	<0.001
**Model 2**						
HbA1c	0.112	0.068 to 0.155	<0.001	0.266	0.172 to 0.359	<0.001
PG	0.002	0.001 to 0.003	0.002	0.006	0.004 to 0.009	<0.001
FPG	0.003	0.002 to 0.004	<0.001	0.005	0.002 to 0.008	0.001

Multiple linear regression models were used to analyze the associations of HbA1c, GP and FPG with CAVI and baPWV.

Model 1: Adjusted for Age (years) and gender (0 = male and 1 = female).

Model 2: Adjusted for Age (years). Gender (0 = male and 1 = female). Smoking (0 = Not and 1 = Yes). Body mass index. Mean arterial pressure. Atherogenic index. Alcohol consumption. Antihypertensive drugs (0 = Not and 1 = Yes). Antidiabetic drugs and lipid lowering drugs (0 = Not and 1 = Yes).

HbA1c glycosylated hemoglobin. PG postprandial glucose. FPG fasting plasma glucose. CAVI cardio-ankle vascular index. baPWV brachial-ankle pulse wave velocity. CI confidence interval. β correlation coefficient.

p statistically significant differences (p < 0.05).

In the multiple linear regression analysis performed on each of the subsamples defined by hyperglycaemic status, a positive association of the three dysglycaemia markers (FPG, PG and HbA1c) with CAVI and baPWV was found only in subjects with type 2 diabetes mellitus (Table 4).

**Table 4:** Multiple regression analysis of HbA1c, PG and FPG with CAVI and baPWV by diabetes status.

	Dependent variable					
	CAVI			baPWV		
	β	95% CI	p	β	95% CI	p
**Type 2 DM** **Model 1**						
HbA1c	0.085	0.024to 0.146	0.006	0.278	0.136 to 0.420	<0.001
PG	0.002	0.001 to 0.003	0.067	0.007	0.003 to 0.011	<0.001
FPG	0.002	0.001 to 0.004	0.040	0.005	0.001 to 0.009	0.022
**Model 2**						
HbA1c	0.102	0.046 to 0.158	<0.001	0.252	0.121 to 0.383	<0.001
PG	0.002	0.001 to 0.004	0.020	0.007	0.003 to 0.010	<0.001
FPG	0.002	0.001 to 0.003	0.014	0.004	0.001 to 0.007	0.048
**Prediabetes** **Model 1**						
HbA1c	0.037	-0.164 to 0.237	0.720	0.023	-0.421 to 0.467	0.920
PG	-0.001	-0.005 to 0.002	0.486	0.001	-0.007 to 0.008	0.889
FPG	0.002	-0.004 to 0.008	0.496	-0.001	-0.013 to 0.013	0.990
**Model 2**						
HbA1c	0.191	-0.001to 0.379	0.053	0.125	-0.294 to 0.544	0.558
PG	0.001	-0.004 to 0.006	0.602	0.001	-0.008 to 0.005	0.743
FPG	0.005	-0.001 to 0.011	0.060	0.004	-0.008 to 0.015	0.524
**Normal glucose** **Model 1**						
HbA1c	-0.165	-0.377 to 0.046	0.125	-0.044	-0.512 to 0.424	0.853
PG	0.001	-0.005 to 0.002	0.872	0.003	-0.007 to 0.013	0.532
FPG	-0.002	-0.010 to 0.006	0.650	0.007	-0.012 to 0.025	0.481
**Model 2**						
HbA1c	-0.063	-0.262to 0.135	0.530	0.125	-0.294 to 0.544	0.558
PG	0.001	-0.003 to 0.006	0.568	0.006	-0.003 to 0.015	0.167
FPG	0.002	-0.006 to 0.010	0.591	0.001	-0.017 to 0.015	0.954

Multiple linear regression models were used to analyze the associations of CAVI and baPWV with HbA1c, GP and FPG.

Model 1: Adjusted for Age (years) and gender (0 = male and 1 = female).

Model 2: Adjusted for Age (years). Gender (0 = male and 1 = female). Smoking (0 = Not and 1 = Yes). Body mass index. Mean arterial pressure. Atherogenic index. Alcohol consumption. Antihypertensive drugs (0 = Not and 1 = Yes). Antidiabetic drugs and lipid lowering drugs (0 = Not and 1 = Yes).

CAVI cardio-ankle vascular index. baPWV brachial-ankle pulse wave velocity. DM diabetes mellitus. HbA1c glycosylated hemoglobin. PG postprandial glucose. FPG fasting plasma glucose. CI confidence interval. β correlation coefficient.

p statistically significant differences (p < 0.05).

**Fig 3:**
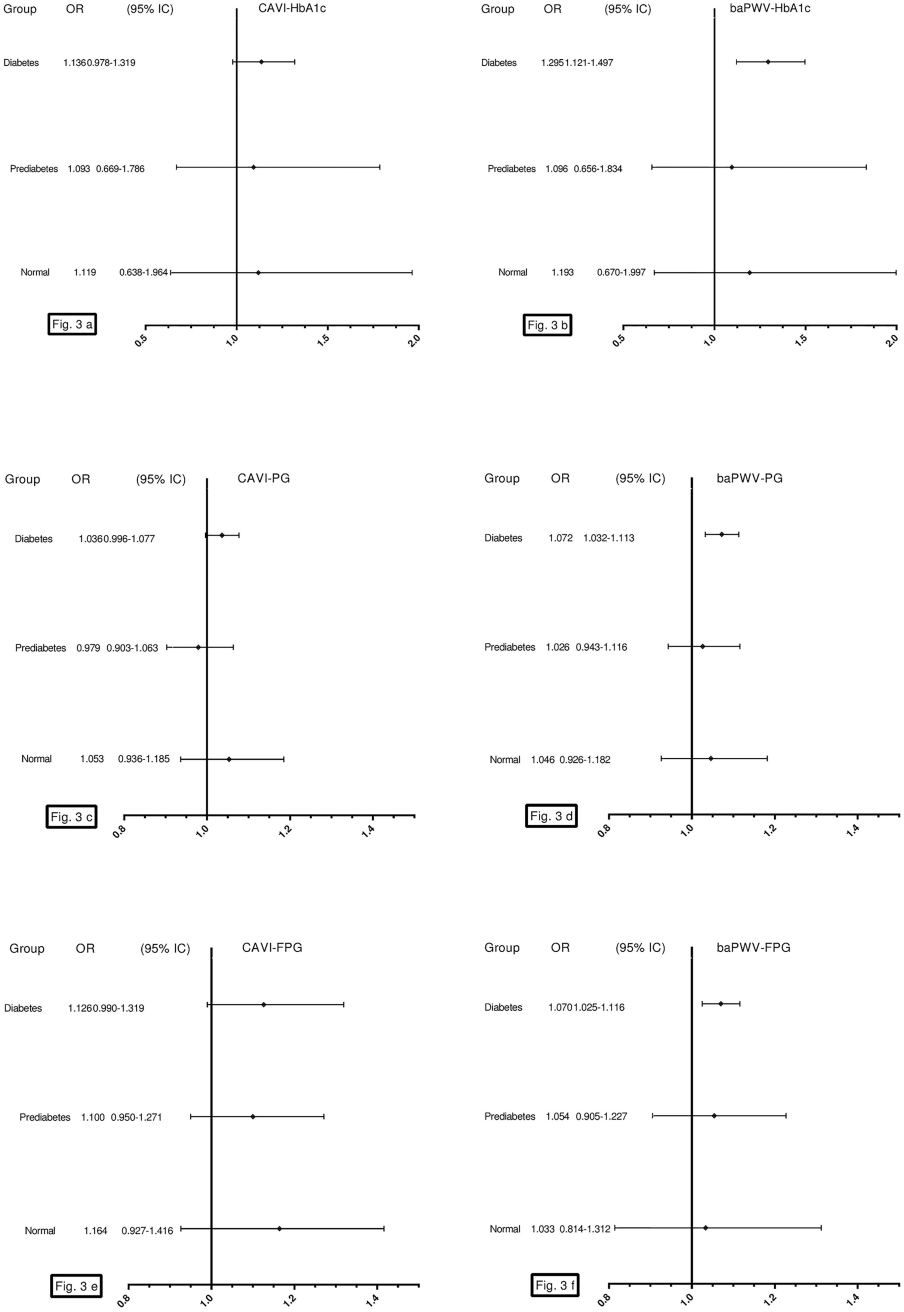
Logistic regression analysis, OR of HbA1c with CAVI (a), of HbA1c with baPWV (b), of PG with CAVI (c), of PG with baPWV (d), of FPG with CAVI (e) and of FPG with baPWV (f). Adjusted for Age (years). Gender (0 = male and 1 = female). Smoking (0 = Not and 1 = Yes). Body mass index. Mean arterial pressure. Atherogenic index. Alcohol consumption. Antihypertensive drugs (0 = Not and 1 = Yes). Antidiabetic drugs and lipid lowering drugs (0 = Not and 1 = Yes). CAVI cardio-ankle vascular index. baPWV brachial-ankle pulse wave velocity. OR odds ratio. CI confidence interval. HbA1c glycosylated hemoglobin. PG postprandial glucose. FPG fasting plasma glucose.

Fig 3 shows the results of the logistic regression analysis after adjusting for the confounding factors used in model 2 of the multiple regression. We only found an association of HbA1c, PG and FPG with baPWV (but not with CAVI), in subjects with type 2 diabetes mellitus: OR = 1.30 with HbA1c and OR = 1.07 with PG and FPG.

## Discussion

The results of this study show a positive association of HbA1c, PG and FPG with CAVI and with baPWV in Caucasians with intermediate cardiovascular risk factors. In the stratified analysis of hyperglycaemic status, this association is only seen in subjects with type 2 diabetes mellitus and is not found in subjects with normal blood glucose concentration or prediabetes. CAVI and baPWV values are higher in diabetic subjects compared to subjects with prediabetes or normal blood glucose. These findings are independent of age, gender, cardiovascular risk factors and use of antihypertensive, hypoglycaemic and hypolipidemic medications.

The review we conducted revealed a controversy with respect to the relationship between dysglycaemia and arterial stiffness. The results we obtained in this study of subjects with intermediate cardiovascular risk factors and normal blood glucose metabolism do not agree with the majority of previously published studies; however, the study population characteristics and adjustment variables were different. In general, there is a positive association of the dysglycaemia indices used, HbA1c [7–9, 36–38], FPG [5, 36, 37] and PG [6], with parameters of arterial stiffness such as baPWV [7, 36], central artery PWV [37, 38], cfPWV [9] or CAVI [6]. Some studies have shown that the association of HbA1c with arterial stiffness is greater than that observed with FPG and PG [9, 37]. This suggests that HbA1c could be a better predictor of arterial stiffness than FPG or GP. In contrast, Zieman SJ et al. [10] did not find an association between HbA1c and arterial stiffness but did find an association with FPG; Liang J et al. [9] did not find an association between either FPG or PG and cfPWV.

In subjects with prediabetes, we did not find an association between any blood glucose measurements and CAVI or baPWV. Our results agree with those found in previous studies that showed that in subjects with IFG [14] or IGT [39], FPG and PG were not associated with the parameters of stiffness used, even after controlling for age, gender and mean blood pressure. No association was found between HbA1c and baPWV in Chinese subjects [13], but this association was found in other studies in which subjects with IFG presented with greater arterial stiffness and baPWV increased linearly with increases in FPG and HbA1c [11–13, 40]. However, many of those studies did not control for factors such as age and blood pressure. According to many authors, the test used for the diagnosis of prediabetes has an effect on arterial stiffness. Accordingly, Li et al. [15] and Xu et al. [41] found that subjects who only presented with IFG did not show greater baPWV, but subjects with IFG and IGT did present with greater arterial stiffness. This suggests that the impact of isolated IFG on arterial stiffness is not as clear as the impact of IGT. Consequently, the relationship between measurements of blood glucose and arterial stiffness in subjects with prediabetes requires further investigation.

These discrepancies between the reported results in different studies may be due to the characteristics of the subjects who were analysed, the different means of measuring arterial stiffness and the use of different adjustment variables. All of these factors increase the difficulty of comparing results between studies. There is a need for prospective studies that adjust for the main variables affecting arterial stiffness to understand the role of different blood glucose measurements on arterial stiffness in subjects with normal blood glucose metabolism or with prediabetes.

Along with the results of this study, some studies have shown that arterial stiffness is greater in subjects with type 2 diabetes mellitus than in control subjects without diabetes [16, 17]. Multiple studies have shown an association between HbA1c levels and increased arterial stiffness measured by baPWV in subjects with type 2 diabetes [39, 42, 43], including in subjects with recent diagnoses of type 2 diabetes mellitus [15]. Likewise, CAVI was significantly higher in hypertensive subjects with type 2 diabetes mellitus compared with healthy and hypertensive groups [44]. However, this relationship has not been shown in all studies; Xu L [37] did not find any association. In a review conducted by Cecelja et al. [19], diabetes was shown to be independently associated with arterial stiffness in 10 out of 19 studies, which represented only 5% of the variation of cfPWV. Thus, glycaemic parameters are likely not the main determinants of arterial stiffness, especially in older and hypertensive patients. The effects of blood glucose on arterial stiffness are masked to a certain extent due to the association of hypertension with diabetes mellitus.

It also must be remembered that the two stiffness measures used are not equivalent, since CAVI measures central and peripheral stiffness, while baPWV is a peripheral stiffness parameter. Likewise, according to the data published by various authors [28, 45–47], CAVI is an arterial stiffness measure independent of arterial pressure at the time of measurement. However, together with age, the factor with the strongest influence upon baPWV is arterial pressure. We therefore consider that the two measures may be complementary. In this study, the mean baPWV value increased gradually as glucose metabolism deteriorated. This did not happen with CAVI, which only showed higher values in the case of subjects with type 2 diabetes mellitus, as can be seen in Table 2. In this same respect, in diabetic individuals, the logistic regression analysis only identified an association between the blood glucose measures and baPWV, but not CAVI. These results may be interpreted as indicating that baPWV alteration possible precedes CAVI elevation in individuals with altered glucose metabolism. In summary, this study is the first performed on a large sample of Caucasian subjects with intermediate cardiovascular risk and involving subjects with normal blood glucose, prediabetes and type 2 diabetes mellitus. We therefore consider that this article contributes new results, for as far as we know, this is the first study to examine this association in a large sample of Caucasian individuals with intermediate cardiovascular risk, using two stiffness measures little used in western populations (particularly CAVI) with the three measures that assess blood glucose metabolism. The results show the positive association of FPG, PG and HbA1c with CAVI and baPWV in the overall sample and in subjects with type 2 diabetes mellitus, after adjusting for the main factors affecting arterial stiffness (aging, hypertension, smoking, dyslipidaemia and pharmacologic treatment) [19]. The adjustment variables, which were not considered in this study, could limit the interpretation of positive findings. However, in the logistic regression analysis, the association in the individuals with type 2 diabetes was only maintained with baPWV, which could suggest that baPWV alteration precedes CAVI alteration in individuals with type 2 diabetes. This disagreement between studies can be partially explained by different arterial stiffness measurements and different study group characteristics (age, sex, race, number and the adjustment variables). Additionally, the measurements used may reflect abnormalities in blood glucose metabolism. Thus, high levels of FPG may represent dysfunction in pancreatic β cells, postprandial hyperglycaemia may be associated with insulin resistance [48], and HbA1c provides a weighted average of blood glucose during the life span of an erythrocyte. These measurements could represent recent changes in diet or treatment [49].

The main limitation of our study is its cross-sectional design, which does not allow us to establish causal relations or the direction of the influence of vascular structure and function parameters on FPG, PG and HbA1c or vice versa. The population in this study was ethnically homogenous, as the study analysed only Caucasian subjects with intermediate cardiovascular risk; thus, the generalizability of our findings could be limited. Finally, glucose intolerance was not achieved with an oral overload of 75 g of glucose, but we were able to determine 2-hour postprandial (after breakfast, lunch and dinner) mean capillary blood glucose levels.

### Conclusions

FPG, PG and HbA1c show associations with CAVI and baPWV, independent confounds in Caucasian adults with intermediate cardiovascular risk. In an analysis of hyperglycaemic status, the association was only maintained in subjects with type 2 diabetes mellitus.

Further longitudinal studies are needed to confirm the relationship between FPG, PG and HbA1c with CAVI and baPWV.

## Supporting information

S1 Database Base con 2233 Base Ploa ONE. (SAV)

## Abbreviations

baPWV: brachial-ankle pulse wave velocity
CAVI: cardio-ankle vascular index
cfPWV: carotid femoral pulse wave velocity
FPG: fasting plasma glucose
HbA1c: glycosylated hemoglobin
IFG: impaired fasting glucose
IGT: impaired glucose tolerance
MARK: MediAte Risk management
PG: postprandial glucose
